# A tiger is not always a satyr: role of male mating behaviour in interspecific mating interactions between *Aedes aegypti* and *Aedes albopictus*

**DOI:** 10.1186/s13071-026-07267-7

**Published:** 2026-03-13

**Authors:** Maisie Vollans, Chenrui Zhang, Catalina Alfonso-Parra, Krijn P. Paaijmans, Simon Gubbins, Michael B. Bonsall, Lauren J. Cator

**Affiliations:** 1 Department of Biology, University of Oxford, Oxford, UK; 2 Department of Life Sciences, Imperial College London, Ascot, UK; 3 Instituto Colombiano de Medicina Tropical, Universidad CES, Sabaneta, Colombia; 4 Max Planck Tandem Group in Mosquito Reproductive Biology, Universidad de Antioquia, Medellín, Colombia; 5 The Center for Evolution & Medicine, School of Life Sciences, Arizona State University, Tempe, AZ USA; 6 Simon A. Levin Mathematical, Computational and Modeling Sciences Center, Arizona State University, Tempe, AZ USA; 7 Wits Research Institute for Malaria, Faculty of Health Sciences, University of the Witwatersrand, Johannesburg, South Africa; 8 The Pirbright Institute, Pirbright, UK; 9 Peter’s College, Oxford, UK

**Keywords:** *Aedes aegypti*, *Aedes albopictus*, Mating ecology, Invasion, Reproductive interference, Interspecific mating, Satyrization, Female choice, Male choice

## Abstract

**Background:**

Interspecific mating between *Aedes aegypti* and *Aedes albopictus*, which act as vectors of a wide range of arboviruses, is predicted to have an important influence on their future distributions and disease control efforts. While interspecific insemination has been documented in a range of laboratory and field studies, rates vary between populations. It has been suggested that the outcome of mating interactions is driven by evolved variation in both the male ability to mate interspecific females and ability of females to resist interspecific males. However, there has been relatively little dissection of the behaviours that underlie interspecific insemination rates.

**Methods:**

We compared insemination rates between sympatric, allopatric, and lab-adapted strains of *Ae. aegypti* (Colombia, Arizona, and Liverpool, respectively) and allopatric and sympatric strains of *Ae. albopictus* (Montpellier and Colombia, respectively)*.* We then used both live observations and high-speed videography to compare intraspecific and interspecific mating interactions.

**Results:**

We found very low rates of interspecific mating across all strains used. Both behavioural observations and high-speed videography suggested that female resistance behaviours were not responsible for low interspecific mating. Interestingly, we documented male *Ae. albopictus* consistently aborting interspecific mating attempts.

**Conclusions:**

This study provides additional evidence for strong pre-copulatory species barriers between *Ae. aegypti* and *Ae. albopictus* and adds male avoidance as a possible mechanism to maintain these barriers. Considering female resistance, the ability of males to overcome that resistance, and male avoidance of interspecific matings together will be important for informing understanding of how we expect interspecific mating rates to vary across populations and respond to selective pressures such as mass-release strategies.

**Graphical Abstract:**

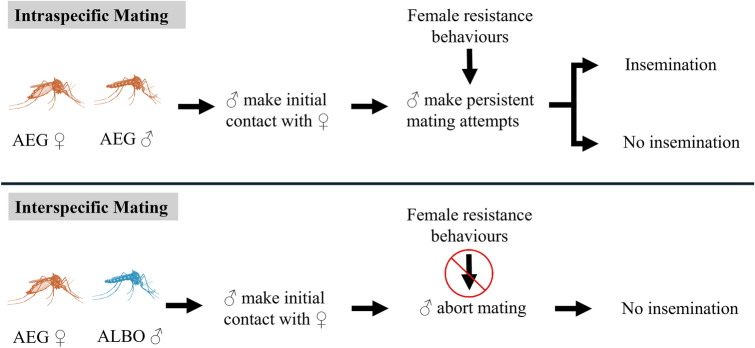

**Supplementary Information:**

The online version contains supplementary material available at 10.1186/s13071-026-07267-7.

## Background

The Yellow Fever mosquito, *Aedes aegypti* and the Asian Tiger mosquito, *Aedes albopictus*, transmit several viruses including those that cause dengue, chikungunya, Zika and yellow fever. Both species have a long history of biological invasion [1, 2] and their contemporary ranges overlap and continue to expand [3].

Interspecific insemination at rates of ~1–3% have been detected between these species in nature [4, 5]. Interspecific mating has asymmetrical costs in these species. Matings between a male *Ae. albopictus* and female *Ae. aegypti* produce no offspring and render the female refractory to subsequent mating attempts. The reciprocal pairing, between male *Ae. aegypti* and female *Ae. albopictus*, also results in no offspring, but females are readily able to remate [4, 6–8]. This asymmetry is thought to be largely due to differences in how these species respond to interspecific male accessory gland proteins (MAGs) and the ability of these to induce refractory behaviour in females [4, 9].

The likelihood of interspecific insemination appears to vary with the genetic background of *Ae. aegypti* and *Ae. albopictus.* Laboratory assessments indicate mating interactions in many populations do not result in any interspecific insemination [10–13], while other populations consistently exhibit varying levels of interspecific insemination [14–18]. The ability to anticipate the likelihood of interspecific insemination in a specific area is important for two key aspects of arbovirus prediction and control. First, the level of interspecific insemination, along with other mechanisms such as resource competition in larvae [19], is an important determinant of between-species competition and influences the overlap and potential for coexistence between these two important vector species [20, 21]. Second, it has been proposed that reproductive interference could be used to suppress *Ae. aegypti* during mass releases of sterile *Ae. albopictus* [10]. Vollans and Bonsall [22] further demonstrated, theoretically, that for *Ae. aegypti* control, where *Ae. aegypti* and *Ae. albopictus* co-occur, reproductive interference can lower the sterile-to-wild *Ae. aegypti* release ratio necessary to achieve population suppression.

There are several proposed barriers to interspecific insemination [13]. Recent work has highlighted differences in male sensory biology [23] and structural incompatibility in genitalia [24]. Heritable variation in both male and female behaviours have also been proposed as key pre-copulatory barriers. Several studies on *Ae. aegypti* populations from the southeastern USA have reported that evolved changes in *Ae. aegypti* females in response to the high costs of interspecific mating are a key determinant of interspecific insemination rates. *Ae. aegypti* females from allopatric populations have been reported to be 0–20% more likely to be inseminated by male *Ae. albopictus* than females from regions where these species are sympatric in controlled laboratory assays [15, 25]. The proposed explanation for this pattern is that females from populations in sympatry evolve costly resistance behaviours to prevent interspecific matings, while allopatric or naïve populations do not evolve or maintain resistance because it is costly [26]. Experimental evolution studies have shown that *Ae. aegypti* females can rapidly evolve to both gain (1–3 generations) and lose (2–4 generations) resistance depending on their exposure to *Ae. albopictus* males [17, 27, 28]. Other studies have highlighted variation in the ability of male *Ae. albopictus* to inseminate *Ae. aegypti* females. For example, *Ae. albopictus* from Brazil were less likely to inseminate *Ae. aegypti* females from multiple locations than males from Vero Beach, FL, USA [18]. It is proposed that the limiting factors relate to the aggressiveness of males and the resistance of females to interspecific mating attempts [17, 18, 27, 28].

Resistance or susceptibility of female *Ae. aegypti* to interspecific insemination has almost exclusively been determined by measuring insemination rates. Interspecific and intraspecific combinations of males and females have been held together for a specified period of time, and resistance has been measured by calculating the proportion of females which have been inseminated [10–18, 29]. There is not a specific threshold by which these populations are classified as susceptible or resistant, rather this distinction is made on the basis of relative insemination within a given experiment. It remains unclear how these laboratory measures reflect the proportion of females being inseminated by interspecific males in the field. Nevertheless, in standardised assays, different populations have repeatable differences in the likelihood of interspecific insemination.

However, the behavioural interactions which underlie variation in inter- and intraspecific insemination rates are rarely addressed. These in-flight interactions are brief, normally lasting <30 s [30]. Harper and Paulson [31] reported that *Ae. aegypti* females did not resist mating attempts by *Ae. albopictus* by kicking the males away or holding males away from their bodies and found that removal of the hindtarsi of females had no effect on interspecific insemination rates. They also reported that in their experiments male *Ae. aegypti* and *Ae. albopictus* ‘rarely attempted to copulate’ with interspecific females [31]. More recently, Zhou et al. [7] compared mating interactions between *Ae. aegypti* males and *Ae. albopictus* females with *Ae. albopictus* males and *Ae. aegypti* females. At high densities, they found that *Ae. aegypti* males attempted to mate with *Ae. albopictus* females more frequently and remained in copula for a shorter duration than *Ae. albopictus* males attempting to mate with *Ae. aegypti* females. Their results suggested that female behaviour was not the determinant of interspecific mating rates. However, further interpretations were limited owing to lack of intraspecific controls. Therefore, despite the suggestion that evolved changes in female mating behaviour are a key determinant of interspecific mating outcomes, there is little documentation of female behaviours that prevent interspecific insemination.

We measured insemination rates of several populations of *Ae. aegypti* and *Ae. albopictus*. We expected female *Ae. aegypti* from strains previously exposed to *Ae. albopictus* (sympatric strains) to be resistant and exhibit lower interspecific insemination rates than those from populations which had not been exposed (allopatric). Further, we expected that these low insemination rates would be associated with higher rates of female resistance behaviours toward interspecific males.

## Methods

### Mosquito rearing

We used three strains of *Ae. aegypti* in our experiments. The Arizona strain of *Ae. aegypti* (AEG_AZ_) originated from a field population in Maricopa County, AZ, USA, which was collected in 2018 by Dr Michael Riehle from the University of Arizona. This strain was kindly provided to Huijben-Paaijmans group and has been maintained in colony since 2019. We used generation F17–F21 in our experiments. This population is believed to be allopatric with *Ae. albopictus* with no historical overlap in distribution. The sympatric Colombian strain of *Ae. aegypti* (AEG_COL_) was collected from ovitraps in Comuna 2 in Medellín, Colombia, a region where *Ae. albopictus* are also present. We used F5–F10 in experiments. The Liverpool strain of *Ae. aegypti* (AEG_LIV_) was originally collected in West Africa and has been maintained in colony since 1938 and has undergone a large unknown number of generations in the lab. We measured interspecific mating rates using two strains of *Ae. albopictus*. The sympatric Colombian strain (ALBO_COL_) was collected in the same city as AEG_COL_: Medellín, Colombia. ALBO_COL_ were collected from a natural site in the Jardín Botánico de Medellín, and the eggs used in this experiment were F2–F3. The allopatric Montpellier strain of *Ae. albopictus* was collected from Montpellier, France, in 2016 (ALBO_MP_) and was provided by Dr Ana Rivero at the University of Montpellier in 2018. The colony has since been maintained in Imperial College London for >20 generations. Strain information is summarised in Table 1.

**Table 1:** Summary of strains used in insemination assays

Species	Strain name	Distribution	Generations used in experiments
*Ae. aegypti*	Arizona	Allopatric	F17–F21
	Colombia	Sympatric	F5–F10
	Liverpool	Lab-adapted	In colony since 1938
*Ae. albopictus*	Colombia	Sympatric	F2–F3
	Montpellier	Allopatric	>F20

Full details can be found in the methods

For all strains, eggs were hatched under a vacuum and provided with ~0.4 mg of crushed fish food (Cichlid gold no. 04328, Hikari, Himeji City, Japan) and placed inside a 27 °C and 70% relative humidity incubator overnight. The following day, first-instar larvae were transferred into 500-ml plastic tubs in groups of 250 larvae and provided diet ad libitum until pupal emergence.

Owing to the number of virgin males and females required for the experiment, several were sexed at the pupal stage, and several at the adult stage. Pupae were sexed under the dissection microscope, using differences in the genital lobe shape to differentiate between sexes [32]. Pupae of the same sex and line were placed into emergence cups in groups of a maximum of 100 pupae. Two cups were placed in a flight cage (22 × 8 × 16 cm plastic containers with an 18 × 6 cm mesh window in the lid) and monitored daily for emergence. Adults were sorted when they were <24 h old, thus, prior to reaching sexual maturity. Upon emergence, sex was determined by morphological differences: females have non-plumose antennae, and males have plumose antennae. Adults were then moved into cages of the same sex and strain. There was a maximum of 200 adults per cage. Adult mosquitoes were provided with a 10% sucrose solution and held at 27 °C and 70% relative humidity on a D12:N12 (with 30 min of dawn/dusk separating each phase) circadian cycle throughout the experiment.

### Insemination assays

Inter- and intraspecific insemination rates were measured by transferring five 4–11-day-old virgin males and five 4–11-day-old virgin females in a 450-ml cup with a mesh lid. Males and females were held together for 1 week and provided with 10% sucrose solution. After 1 week of exposure to virgin males, all females from each replicate cup were dissected to determine whether insemination had occurred. Females were briefly immobilized on ice, their spermathecae dissected, and the presence/absence of sperm determined using a compound microscope [33].

We conducted two insemination assays (Table 2). In the first experiment, we compared interspecific insemination rates of female AEG_AZ_ and female AEG_COL_ held with male ALBO_COL_ (♀AEG_AZ_ × ♂ALBO_COL_; ♀AEG_COL_ × ♂ALBO_COL_) with intraspecific mating rates within and between the *Ae. aegypti* strains (♀AEG_AZ_ × ♂AEG_AZ_; ♀AEG_AZ_ × ♂AEG_COL_; ♀AEG_COL_ × ♂AEG_COL_; ♀AEG_COL_ × ♂AEG_AZ_) for a total of six combinations. In the second experiment, we explored the effect of *Ae. albopictus* strain on interspecific insemination rates. We compared insemination rates when female AEG_COL_, AEG_AZ_ and AEG_LIV_ were held with males from their own strain (♀AEG_AZ_ × ♂AEG_AZ_, ♀AEG_COL_ × ♂AEG_COL_, ♀AEG_LIV_ × ♂AEG_LIV_) to insemination rates when these females were held with ALBO_COL_ and ALBO_MP_ (Table 1). We measured five replicate cups of five females for each mating combination except those with ALBO_COL_ males in the second insemination experiment, where we conducted three replicate cups owing to insufficient males.

**Table 2:** Insemination rates recorded for crosses between different strains of *Ae. aegypti* and *Ae. albopictus*

Experiment number	Female species and strain	Male species and strain	No. replicate cups (no. females dissected per treatment)	Proportion inseminated [Mean (Min, Max)]
1	*Ae. aegypti* AZ	*Ae. aegypti* AZ	5 (24)	0.63 (0.40, 0.80)
1		*Ae. aegypti* COL	5 (26)	0.70 (0.50, 1.00)
1		*Ae. albopictus* COL	5 (23)	0 (0.00,0.00)
1	*Ae. aegypti* COL	*Ae. aegypti* AZ	5 (23)	0.91 (0.75, 1.00)
1		*Ae. aegypti* COL	5 (25)	0.93 (0.80, 1.00)
1		*Ae. albopictus* COL	5 (24)	0 (0.00,0.00)
2	*Ae. aegypti* COL	*Ae. aegypti* COL	5 (25)	0.76 (0.40, 1.00)
2		*Ae. albopictus* COL	3 (14)	0.13 (0.00, 0.20)
2		*Ae. albopictus* MP	5 (24)	0 (0.00,0.00)
2	*Ae. aegypti* AZ	*Ae. aegypti* AZ	5 (24)	0.77 (0.60, 1.00)
2		*Ae. albopictus* COL	3 (15)	0 (0.00,0.00)
2		*Ae. albopictus* MP	5 (26)	0, (0.00,0.00)
2	*Ae. aegypti* LVP	*Ae. aegypti* LVP	5 (25)	0.83 (0.75, 1.00)
2		*Ae. albopictus* COL	3 (14)	0 (0.00,0.00)
2		*Ae. albopictus* MP	5 (24)	0 (0.00,0.00)

The realised sample sizes deviate from the planned five females per replicate owing to mortality or unclear dissection (number of females) or two instances of an extra female being added to the cup. *AZ *Arizona, *COL *Colombia, *MP *Montpellier, *LIV *Liverpool

### Behavioural observations

To investigate the male and female behaviours underlying differences in insemination rates observed in interspecific and intraspecific combinations, we observed interactions between males and females from the same strain of *Ae. aegypti* (♀AEG_AZ_ × ♂AEG_AZ_; ♀AEG_COL_ × ♂AEG_COL_; ♀AEG_LIV_ × ♂AEG_LIV_) and compared these interactions with interactions between females from the same strains and male ALBO_MP_ (♀AEG_AZ_ × ♂ALBO_MP_; ♀AEG_COL_ × ♂ALBO_MP_; ♀AEG_LIV_ × ♂ALBO_MP_). Further details of the crosses are in the supplementary information (Additional File 1, Supplementary Table S1). We transferred four 7–10 day old virgin males from the same strain into a 30-cm^3^ clear plastic cage placed on a dark mounting. We used multiple males to emulate more natural conditions, as swarms can form with as few as two males [34, 35]. Males were allowed to habituate to the cage for 5 min. Trials started when a single 7–10-day-old virgin female was released into the cage and ended after the first copula finished or after 4 min had lapsed (whichever came first). If a copula was initiated before 4 min elapsed, we continued recordings until the copula broke apart. After the copula had broken apart, the female was immediately removed from the cage to ensure that any insemination was a result of the observed copula.

We recorded the total number of mating attempts, the timing of all mating attempts, whether a copula formed, the start and stop time of the copula and the type(s) of mating resistance behaviour exhibited. Resistance behaviours were classified following Cator and Harrington [36] and Aldersley and Cator [30] and included kicks and holds. Briefly, a kick was counted when the female physically dislodged the male with her legs and a hold was counted when the female used her legs to prevent the male from moving into copula position or tilted her abdomen to prevent genital contact. We used these data to calculate attempt period (the length of time attempts were made over a trial) and attempt rate (the number of mating attempts scaled by the attempt period). Females which were observed to copulate were briefly immobilized on ice and their spermathecae were dissected under a dissection microscope. We examined the spermathecae under a compound microscope to determine the presence or absence of sperm.

### High-speed video

To examine fine scale mating behaviour, we recorded high-speed video (Phantom Miro 310, Vision Research, Wayne, NJ, USA) of interactions between tethered female AEG_AZ_ presented with either male AEG_AZ_ or ALBO_MP_. We also recorded interactions between female ALBO_MP_ and male ALBO_MP_. We recorded interactions of ten pairs for each combination. Following Aldersley and Cator [30], individual 5–10-day-old virgin females were cold anaesthetised and attached to a ~20-mm strand of human hair connected to an insect pin (4 cm, size 02, Bohemia^®^, Czech Republic) using adhesive (Nailene, Pacific World Inc., Aliso Viejo, CA, USA). Tethered females were positioned on a stand in the centre of a 30-cm^3^ cage. The cage was positioned on top of a heating plate which was covered with a worn t-shirt to provide host cues (25 ± 5.0 °C, 28.1 ± 2.0% RH monitored using an EasyLog USB data logger, Lascar Electronics Ltd, Whiteparish, Wiltshire, UK). Females were given a small piece of cotton wool to hold during a 1–3 min recovery period. We stimulated female flight by removing the cotton wool, which released tarsal inhibition, and gently blowing on females. We released individual 5–10-day-old virgin males into the arena. We used a configurable software trigger (PCC, Vision Research, Wayne, NJ, USA) to initiate recordings when the male first attempts to make physical contact with the tethered female. The trigger allowed us to record the 5 s prior and 23 s following the first contact between the male and female, resulting in a total of 28 s of video recording at 1000 fps and a frame size of 5–7 cm. We recorded the duration of the attempt(s) and whether an attempt ended in a rejection (kicks/holds) or copula.

### Data analysis

All analyses were run in R version 4.3.1 (R Core Team, 2023). Data was processed using dplyr [37] and tidyr [38], and all graphs were created using ggplot2 [39]. The package lme4 [40] was used to fit linear and generalised linear mixed-effects models (GLMM). The package MASS [41] was used to display how well the error distribution fits the response variable of interest, conduct likelihood ratio tests and produce *P*-values for fixed effects.

Explanatory variables with a significant effect (*P* ≤ 0.05) were reported in the results section, and those without a significant effect were excluded from models and included in the supplementary information (Additional File 1, Supplementary Tables S2–S5).

For the insemination experiment, we assessed the effect of mating combinations on insemination rates using GLMMs with a binomial response variable and logit link function. We assessed the effect of mating type (interspecific/intraspecific) across all treatments by including it as a fixed effect. We included the fixed effects of male strain (AEG_COL_/AEG_AZ_), female strain (AEG_COL_/AEG_AZ_) and their interaction on the likelihood of insemination in *Ae. aegypti* combinations. In all cases, the replicate cup was included as a random effect to account for repeated measures. When an explanatory variable with a significant effect on the response variable had two levels, lmerTest was used to produce summary statistics of the directional effect [42]. However, where an explanatory variable with a significant effect had more than two levels, a Tukey test was conducted to examine the pairwise differences between groups, using the emmeans package [43]. In instances in which a group of data consisted of all zeros (which occurred when no females in a combination were inseminated), we applied a Pearson’s chi-squared test. This is because GLMMs are inappropriate in these instances as the poisson distribution and binomial distribution cannot be defined from zeros alone.

GLMMs were also applied to analyse the mating behaviour data. To assess the fixed effects of mating type (interspecific/intraspecific), male strain (AEG_COL_/AEG_AZ_/AEG_LIV_/ ALBO_MP_) and female strain (AEG_COL_/AEG_AZ_/AEG_LIV_) on whether or not a mating attempt, copula, kick, hold or insemination occurred, we fit models using a binomial distribution. GLMMs exploring effects on latency to first attempt and total attempts were fit with a normal distribution. The model investigating the total number of attempts was fit with a poisson distribution. The model exploring copula duration was fit with a gamma distribution. We also included timing block as a fixed effect in all models, as *Aedes* activity levels vary predictably throughout the day [44–46]. Details of partitioning timing blocks are detailed in the supplementary information (Additional file 1). For all models, we additionally included experimenter and experimental block as random effects. As each experimenter conducted multiple experimental blocks, the random effect of experimental block was nested within the experimenter. To estimate effect sizes, we used Cohan’s *d* [47]. When necessary, we used weighted Akaike information criterion (AIC) in model selection (for further details, see Additional File 2).

Pearson’s chi-squared tests were used to assess the effect of treatments on whether or not behaviours occurred and for counts of behaviours in interactions. Furthermore, when continuous data did not fit the normal distribution or the gamma distribution, we conducted simple statistics: Wilcoxon rank-sum test (when the explanatory variable two levels) or the Kruskal–Wallis rank sum test (when the explanatory variable had more than two levels).

For high-speed video recordings, we assessed the effect of mating type (interspecific/intraspecific) on the duration of the attempt using a Wilcoxon rank-sum test. To look at differences between specific combinations, we used a Kruskal–Wallis rank sum test. A Pearson’s chi-squared test was used to compare the occurrence of copulas and resistance behaviours across combinations.

All data are supplied in Additional File 3.

## Results

### Insemination assays

In both insemination experiments, interspecific insemination was rare. In the first insemination experiment, no *Ae. aegypti* females from either strain (AEG_AZ_ or AEG_COL_) were inseminated by *Ae. albopictus* males (ALBO_COL_), and insemination rates were significantly higher in intraspecific crosses than interspecific crosses (Table 1, Pearson’s chi-squared, *χ*^2^ = 75.62, df = 1, *P* < 0.001). Females were equally as likely to be inseminated by males from either the same intraspecific strain or a different intraspecific strain (likelihood ratio test: *χ*^2^ = 0.54, df = 1, *P* = 0.82). Similarly, male strain had no impact on the likelihood of insemination in intraspecific crosses (likelihood ratio test: *χ*^2^ = 0.19, df = 1, *P* = 0.67). However, the origin of female *Ae. aegypti* influenced the likelihood of insemination by *Ae. aegypti* males (likelihood ratio test: *χ*^2^ = 9.82, df = 1, *P* < 0.01). A Tukey-adjusted pairwise comparison showed that AEG_AZ_ females were inseminated less frequently than AEG_COL_ females (estimate = −1.73, SE = 0.602, *z* = −2.88, *P* = 0.0039).

We observed similar trends in our second experiment, where we used an additional strain of *Ae. aegypti* (AEG_LIV_) and *Ae. albopictus* (ALBO_MP_) (Table 1). Interspecific insemination among these strains was also rare, with a total of two females inseminated out of 117 interspecific crosses. Thus, significantly fewer *Ae. aegypti* females were inseminated by *Ae. albopictus* males than *Ae. aegypti* males (likelihood ratio test: *χ*^2^ = 64.14, df = 1, *P* < 0.001). In this experiment, there was no difference in the proportion of females inseminated by *Ae. aegypti* males from different strains (likelihood ratio test: *χ*^2^ = 0.74, df = 1. *P* = 0.69).

### Behavioural observations

**Fig. 1:**
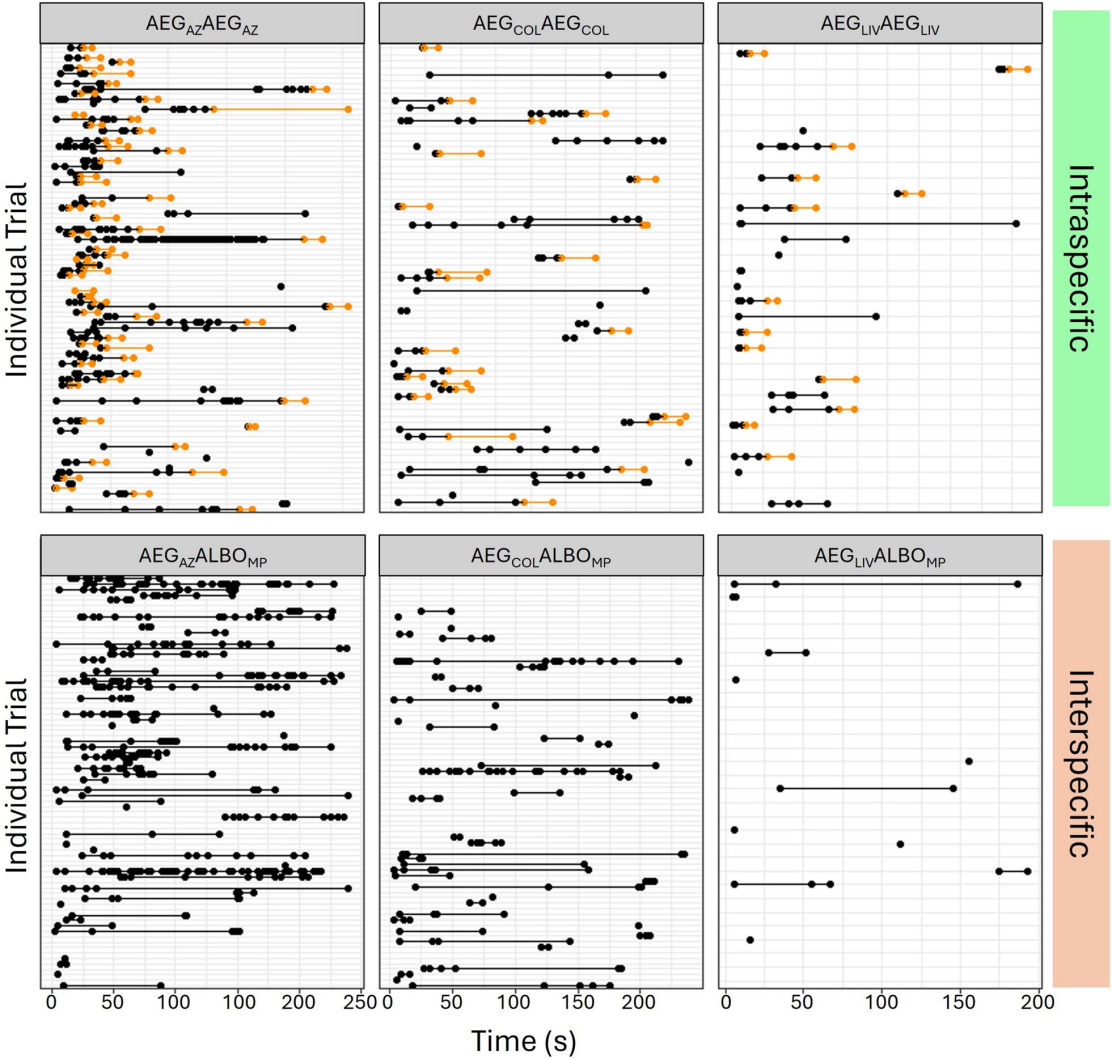
Visualisation of individual mating interactions between female *Ae. aegypti* from three strains (*AZ *Arizona, *COL *Colombia, *LIV *Liverpool) when paired with males from their own strain (intraspecific pairings) or with male *Ae. albopictus* (*MP *Montpellier). Black dots and lines indicate attempts to mate with females. Orange dots and lines indicate data recorded from copulas. The absence of lines or dots in a trial indicates that no attempts were made

Given that both the allopatric ♀AEG_AZ_ and highly lab-adapted ♂AEG_LIV_ exhibited low interspecific insemination rates, we further investigated the interactions between these strains. We observed 372 mating trials (Fig. 1). Across combinations, the probability of a mating attempt occurring differed by female strain (likelihood ratio test: *χ*^2^ = 17.77, df = 2, *P* < 0.001), male strain (likelihood ratio test: *χ*^2^ = 10.80, df = 3, *P* = 0.01) and timing block (likelihood ratio test: *χ*^2^ = 4.80, df = 1, *P* = 0.03). A Tukey-adjusted pairwise comparison found that mating attempts across all combinations were more likely to occur in the morning (09:30–12:00) than afternoon (12:00–15:45) (estimate = −0.62, SE = 0.29, *z* = 2.17, *P* = 0.03). Furthermore, males of both species were more likely to attempt to mate with ♀AEG_AZ_ (Tukey-adjusted pairwise comparison: estimate = 2.16, SE = 0.54, *z* = 4.04, *P* < 0.001) or ♀AEG_COL_ (Tukey-adjusted pairwise comparison: estimate = 1.36, SE = 0.50, *z* = 2.72, *P* = 0.018) than ♀AEG_LIV_. ♂AEG_LIV_ were more likely to attempt to mate with females than both ♂AEG_COL_ (Tukey-adjusted pairwise comparison: estimate = 1.93, SE = 0.68, *z* = 2.82, *P* = 0.03) and ♂ALBO_MP_ (Tukey-adjusted pairwise comparison, estimate = 1.78, SE = 0.58, *z* = 3.06, *P* = 0.01). However, intraspecific (♀AEG_COL_ × ♂AEG_COL_;♀AEG_AZ_ × ♂AEG_AZ_;♀AEG_LIV_ × ♂AEG_LIV_) and interspecific (♀AEG_COL_ × ♂ALBO_MP_;♀AEG_AZ_ × ♂ALBO_MP_;♀AEG_LIV_ × ♂ALBO_MP_) pairs were equally likely to include a mating attempt (likelihood ratio test: *χ*^2^ = 0.24, df = 1, *P* = 0.10).

**Fig. 2:**
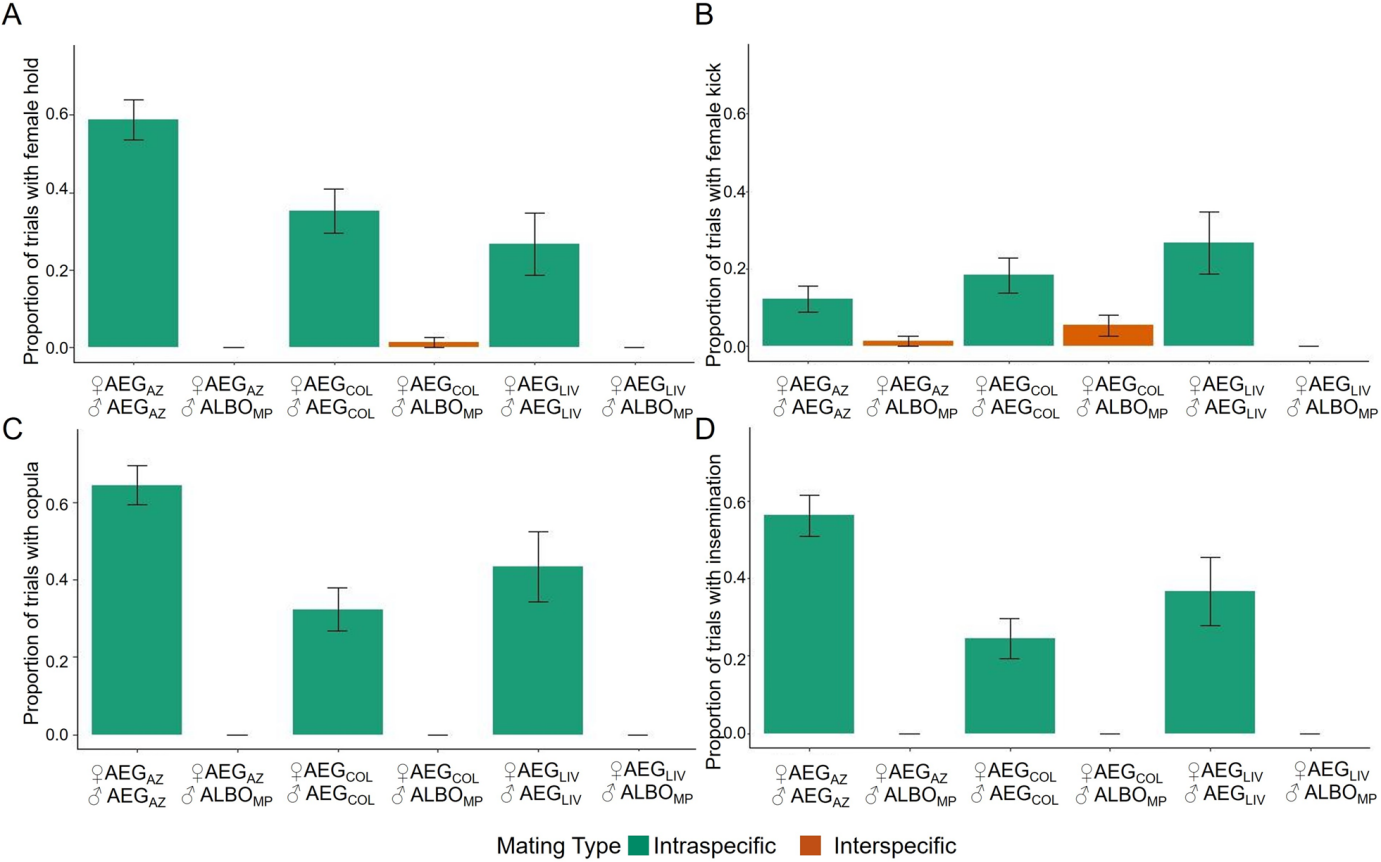
**A** Proportion of trials in which a female was observed to hold the attempting male away. **B** Proportion of trials in which a female kicked the male away during an attempt. **C** Proportion of trials ending in a copula. **D** Proportion of trials ending with insemination. *Bars* represent ± 1 SE

In trials in which attempts occurred, there was a significant effect of the cross type (interspecific/intraspecific) on the duration of the attempt period (Fig. 2a, likelihood ratio test: *χ*^2^ = 12.25, df = 1, *P* < 0.001). *Ae. aegypti* males attempted to mate with *Ae. aegypti* females for a longer period of time than *Ae. albopictus* males attempted to mate with *Ae. aegypti* females (Tukey-adjusted pairwise comparison: estimate = 0.0068, SE = 0.0020, z = 3.45, *P* < 0.001). However, the effect size was small (*d* =  −0.0074).

We found that *Ae. aegypti* females almost exclusively performed resistance behaviours towards males from their own strain. Only one female was observed to hold an interspecific male away from her abdomen, and females were only observed to kick interspecific males away on three occasions. Thus, females were significantly more likely to hold (Fig. 2b, Pearson’s chi-squared test, *χ*^2^ = 89.28, df = 1, *P* < 0.001) and kick away intraspecific males than interspecific males (Fig. 2c, likelihood ratio test: *χ*^2^ = 21.66, df = 1, *P* < 0.001).

Intraspecific crosses were significantly more likely to result in a copula (Pearson’s chi-squared test, *χ*^2^ = 116.61, df = 1, *P* < 0.001) and insemination (Pearson’s chi-squared test: *χ*^2^ = 92.35, df = 1, *P* < 0.001) than interspecific crosses (Fig. 2C, D). We did not observe copulas in any of the interspecific treatments. Within intraspecific crosses, the strain impacted the likelihood of a copula forming (likelihood ratio test: *χ*^2^ = 9.07, df = 2, *P* = 0.011) and insemination occurring (likelihood ratio test: *χ*^2^ = 6.34, df = 2, *P* = 0.042), with copulas more likely to form (Tukey-adjusted pairwise comparison: estimate = 1.39, SE = 0.46, *z* = 3.00, *P* = 0.0076) and insemination more likely to occur (Tukey-adjusted pairwise comparison: estimate = 1.25, SE = 0.49, *z* = 2.54, *P* = 0.030) with ♀AEG_AZ_ × ♂AEG_AZ_ crosses than the ♀AEG_COL_ × ♂AEG_COL_ crosses. There was a positive correlation between the formation of a copula and insemination occurring (Pearson’s product-moment correlation: cor = 0.911, *t* = 42.12, df = 365, *P* < 0.001).

### High-speed videography

**Fig. 3:**
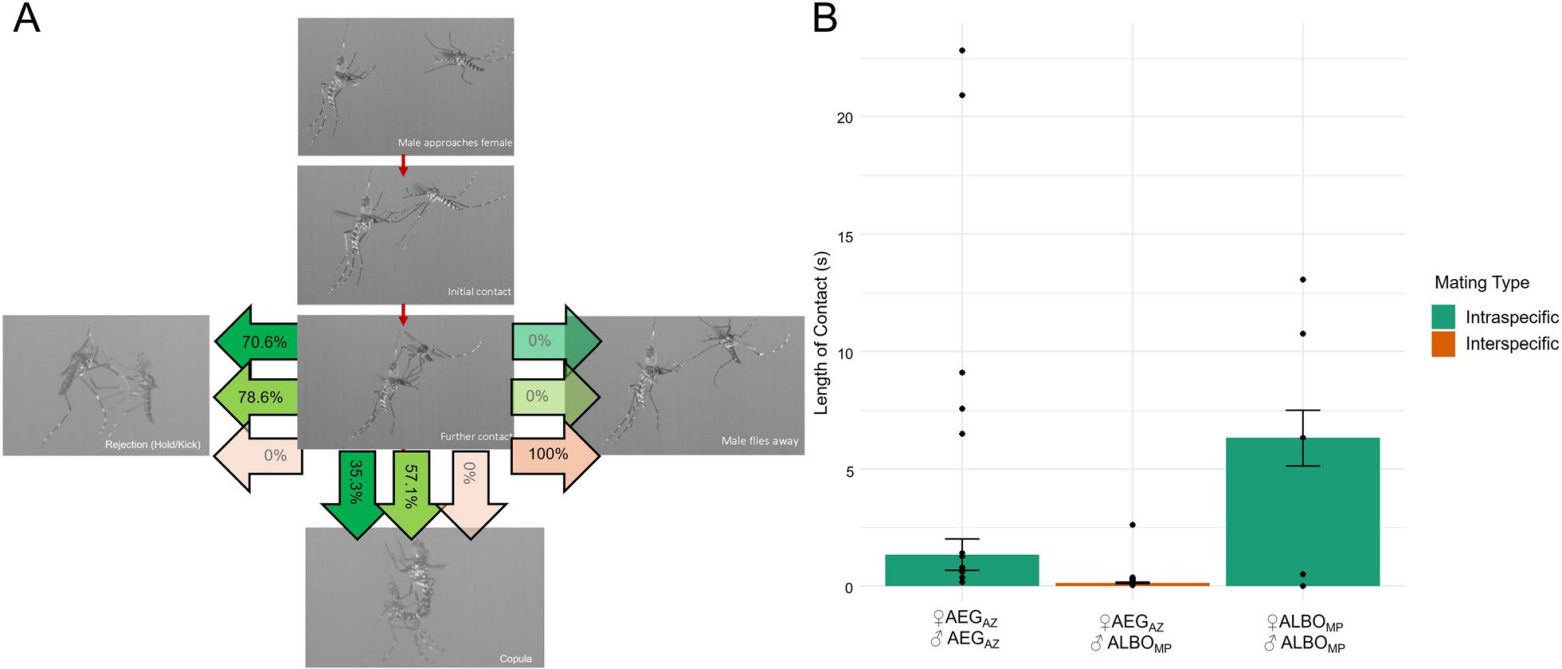
Pre-copulatory interactions between *Ae. albopictus* and *Ae. aegypti.* Males initially approached females with a similar regularity as intraspecific males (**A**). However, once contacting the female, the interaction was much shorter than those observed in intraspecific interactions (**B**) and terminated when the male flew away. Green arrows represent intraspecific combinations (dark green—♀AEG_AZ_ × ♂AEG_AZ_, *n* = 10 pairs, 17 interactions; light green—♀ALBO_MP_ × ♂ALBO_MP_ *n* = 10 pairs, 14 interactions). Orange arrows represent interspecific combination of ♀AEG_AZ_ × ♂ALBO_MP_ (*n* = 10 pairs, 17 interactions).* Bars* in **B** represent ± 1 SE

We captured 48 interactions across 30 pairs (10 pairs ♀AEG_AZ_ × ♂AEG_AZ,_ 10 pairs ♀ALBO_MP_ × ♂ALBO_MP,_ 10 pairs ♀AEG_AZ_ × ♂ALBO_MP_). We observed that all ALBO_MP_ males made brief initial contact with AEG_AZ_ females with their fore- and midtarsi, sometimes followed by further exploratory contact, and then terminated the attempt and flew away (Fig. 3, Additional File 4: Supplementary Video S1). This contact period was shorter than when either ALBO_MP_ or AEG_AZ_ approached females from their own species (Supplementary Video S2, Wilcoxon rank sum test: *W* = 258, df = 2, *P* < 0.001). Almost all of the interspecific interactions (94.12%, *n* = 17) were less than 0.5 s long, while only 17.64% (*n* = 17) of intraspecific interactions were less than 0.5 s long. Both AEG_AZ_ and ALBO_MP_ females were significantly more likely to exhibit resistance behaviour when attempts were made by a male from their own species (Fig. 3, Pearson’s chi-squared test: *χ*^2^ = 6.83, df = 1, *P* = 0.0090). As in the free flight observations, females did not resist interspecific males (0 out of 17 interactions). However, resistance was observed in 74.20% of intraspecific interactions (23/31): 70.60% of interactions between *Ae. aegypti* (12/17) and 78.60% (11/14) of interactions between *Ae. albopictus* (Additional File 5: Video S1). No copulas formed in interspecific crosses, but occurred in both intraspecific interactions (Fig. 3**,** Pearson’s chi-squared test: *χ*^2^ = 8.76, df = 1, *P* = 0.0031).

## Discussion

We observed very low rates of interspecific insemination across combinations of three strains of *Ae. aegypti* and two strains of *Ae. albopictus* (Table 2). While we had expected females from the sympatric AEG_COL_ strain to have evolved resistance to interspecific insemination, we did not expect to observe low mating rates between allopatric AEG_AZ_ or lab-adapted AEG_LIV_ females and *Ae. albopictus* males. We had made this prediction because the AEG_AZ_ was unexposed to *Ae. albopictus* and, thus, had not encountered selective pressure to avoid interspecific insemination, and AEG_LIV_ has been maintained for hundreds of generations in the absence of this pressure [48]. If the evolution of this type of discrimination is costly, as has been shown in other strains of *Ae. aegypti* [27], then we would have expected AEG_AZ_ to have not evolved it in the first place and AEG_LIV_ to have rapidly lost this trait.

Methodological differences are one explanation for this discrepancy. In our experiments, groups of ten (five males, five females) were held together for a 1-week period, whereas several other studies have used larger populations and longer durations. For example, Bargielowski and Lounibus [17], used groups of 300 for a 3-week period. It is possible that if we extended our experiments we might have seen more ‘susceptibility’ to interspecific insemination. However, the instance where we observed any interspecific mating was in the sympatric, not the allopatric *Ae. aegypti* population, which is the opposite of where we would have expected to observe this type of effect.

Alternatively, it may be that in the populations used here, female rejection is not the key mechanism preventing interspecific insemination. Live behavioural observations supported the findings of the insemination assays. No copulas were recorded in any of the interspecific treatments. These data further revealed that while all three strains of *Ae. aegypti* were able to readily reject males of their own species, interspecific males rarely received rejection behaviours (Fig. 2). Out of 181 documented interspecific interactions, only four involved a female *Ae. aegypti* kicking or holding an *Ae. albopictus* male away. High-speed recording supported this initial observation. ♀AEG_AZ_ did not kick or hold ♂ALBO_MP_ away, whereas more than 70% of intraspecific matings attempts in both ♀AEG_AZ_ and ♂AEG_AZ_ ended with one of these types of rejection. These observations are aligned with those reported by both Harper and Paulson [31] and Zhou et al. [7] and additionally demonstrate that the lack of female rejection is specific to interspecific interactions. Thus, populations that would have been classified as ‘resistant’ using interspecific insemination rates did not demonstrate any resistance behaviours. Evidence suggests that both acoustic [23] and chemical cues [49] may be used by males to identify intraspecific females. Our experiments were not designed to test for the role of these cues, but it is interesting to note that all males aborted attempts after tarsal contact. Nijhout and Craig [49] reported that chemoreception in the first two segments of the fore and midtarsi appeared to underlie similar behaviours from male *Ae. albopictus* (VN and POONA strains) presented with *Ae. aegypti* females (ROCK) [49].

In our experiments, allopatric male *Ae. albopictus* exhibited a behaviour in which they rapidly flew away from interspecific females after a very brief contact. This type of ‘touch and go’ behaviour was not observed in the recording of intraspecific pairs. It seems that, unlike the observations of strains from Florida, USA in which *Ae. albopictus* have been reported to inseminate *Ae. aegypti* [31], these males terminated mating interactions quickly. The term ‘satyrization’ was first used by Ribeiro and Spielman [50] and is defined as ‘the act of mating with a female of an incompatible species’. The term is a reference to the satyrs of Greek mythology which were part-human part-animal male characters often featured chasing and seducing nymphs and human females [51]. While not part of the scientific definition, this historical root may erroneously lead to the impression that interspecific mating is always controlled by the aggressiveness of males and the choosiness of females. It appears that the ‘satyr’, *Ae. albopictus*, may also use pre-copulatory mechanisms to avoid interspecific mating.

Given the complexity of the natural history of these two species and the complexity of the behavioural mechanisms underlying interspecific insemination, perhaps it is not surprising that the likelihood of interspecific insemination varies by population [5, 10–13, 15–18]. *Ae. aegypti* and *Ae. albopictus* originated from separate continents, Africa and Asia, respectively, and have gone on to invade large geographical areas. This means that much of their overlapping distribution includes areas in which they are both invasive [20]. They rarely invade these environments simultaneously, and thus, there is not only variation in the environmental conditions in which interspecific mating interactions occur, but also variation in the timing of the relative invasion of these two species at a given location. Existing theory suggests that differences in intraspecific encounter rates from the core of a species range to the leading edge will select on behavioural traits [52, 53] and that reproductive interference will have the greatest impact on the rarer species at a given location [54]. In addition to the duration of sympatry and order of invasion, it seems likely that resistance to interspecific insemination may evolve via several different pre- and postcopulatory barriers [13]. While data from some populations suggests that female behaviour is most important [17, 25], we, and others, have also observed differences in male mating behaviour [7, 31]. This redundancy means it is possible that all, none, or several of these barriers may exist in varying degrees across the species range.

While our use of field derived strains and standardised assays was informative and highlights variation among populations, future work on natural populations is needed to accurately capture natural variation and plasticity in these behaviours. Bargielowski et al. [5] documented interspecific insemination in four geographically distinct field populations with varying durations of overlap and arrival order. While the number of sites was not sufficient to analyse the effect of relative arrival time, future work could apply this type of approach combined with detailed behavioural phenotyping to test evolutionary hypotheses.

## Conclusions

We would expect important phenomena such as reproductive interference [20, 21] to vary widely in strength across populations and locations. The displacement of mating traits is an important potential outcome of reproductive interference [55]. It appears multiple pre- and post-copulatory mating traits determine, and may be affected by, reproductive interference in these species. Further, clarifying the reproductive behaviours and mechanisms in play at a particular site will be important for predicting not only the efficacy of sterile male releases but also the evolutionary responses to these releases. For example, the evolved response of *Ae. aegypti* populations to the release of sterile *Ae. albopictus* [10] will depend on the mechanisms underlying rates of interspecific insemination. Future work systematically testing hypotheses about the propensity for and response of populations to reproductive interference could provide an overarching theoretical framework to explain and predict variation in interspecific mating rates. We suggest that the design of these studies should allow for roles of both male and female biology in their evolution and characterise the specific mechanisms underlying patterns of insemination.

## Supplementary Information

Additional file 1: Text S1. Tables S1-S5, includes experimental blocking for behavioural observations and statistical tables.

Additional file 2: Text S2. Additional methods.

Additional file 3: Dataset S1. Data from Insemination Assays, Behavioural Observations, High-Speed Videography experiments.

Additional file 4: Video S1. High-speed footage of mating attempt between male *Ae. albopictus* (MP) and female *Ae. aegypti* (AZ).

Additional file 5: Video S2. High-speed footage of mating attempt between male *Ae. albopictus* (MP) and female *Ae. albopictus* (MP).

## Data Availability

All data generated or analysed during this study are included in this published article and its supplementary information files.
