# Supplementary material for: A tiger is not always a satyr: role of male mating behaviour in interspecific mating interactions between Aedes aegypti and Aedes albopictus
Source: Parasit Vectors. 2026 Mar 13;19:238. doi: 10.1186/s13071-026-07267-7 (PMC13227695; doi:10.1186/s13071-026-07267-7)
Supplement: Supplementary file 1 — Additional file 1: Text S1. Tables S1-S5, includes experimental blocking for behavioural observations and statistical tables. [file 13071_2026_7267_MOESM1_ESM.pdf]

Supplementary Information – Tables

	Experimental Block						
Cross	1	2	3	4	5	6	Total
AEG_{AZ} AEG_{AZ}	10	30	30	10	-	10	90
$AEG_{AZ}ALBO_{MP}$	30	30	-	6	-	10	76
$AEG_{COL}AEG_{COL}$	30	11	-	10	10	10	71
$AEG_{COL}ALBO_{MP}$	30	19	-	6	10	10	75
$AEG_{LIV}AEG_{LIV}$	-	-	-	10	10	10	30
$AEG_{LIV}ALBO_{MP}$	-	-	-	10	10	10	30

Table S1 – summary of the number of replicates per experimental block in the Behavioural Observations experiment, for each cross. One replicate is defined as one cage observation. Crosses are female-male, such that AEG_{AZ} $ALBO_{MP}$ represents AEG_{AZ} females crossed with $ALBO_{MP}$ males.

Response Variable	Distribution	Female Strain			Male Strain			Timing Block			Female Strain* Timing Block			Male Strain * Timing Block		
		LRT	df	*p*	LRT	df	*p*	LRT	df	*p*	LRT	df	*p*	LRT	df	*p*
Mating Attempt Occurrence	Binomial	17.767	2	0.000139	10.802	3	0.0128	4.7962	1	0.0285	0.336	1	0.562	1.192	2	0.551
Latency to First Mating Attempt	Gamma	1.657	2	0.437	-	-	-	-	-	-	1.498	1	0.221	17.145	2	0.000189
Female Kick	Binomial	2.804	2	0.246	23.439	3	3.270e-05	0.1003	1	0.7515	1.301	1	0.254	2.220	2	0.323
Attempt Period	Gamma	3.693	2	0.158	13.151	3	0.00432	0.0707	1	0.790	0.0847	1	0.771	1.259	2	0.533
Attempt Rate	Gamma	1.565	2	0.457	4.0171	3	0.260	0.795	1	0.373	2.760	1	0.0967	3.406	2	0.182
Effortful Attempt	Binomial	2.866	2	0.239	159.180	3	< 2.2e-16	2.878	1	0.0898	1.490	1	0.222	1.325	2	0.516

Table S2 - outputs of the Likelihood Ratio Tests conducted on all generalised linear mixed effects models examining the explanatory variables male strain, female strain, and their interaction with timing block. These models include data from both conspecific and heterospecific crosses.

Response Variable	Distribution	Cross			Timing Block			Cross*Timing Block		
		LRT	df	*p*	LRT	df	*p*	LRT	df	*p*
Copula Formation	Binomial	9.0711	2	0.0107	4.0498	1	0.0442	0.749	1	0.387
Insemination Occurrence	Binomial	6.338	2	0.0421	4.298	1	0.0382	1.5145	1	0.219
Female Hold	Binomial	3.361	2	0.186	0.202	1	0.653	0.873	1	0.350
Copula Duration	Gamma	5.598	2	0.0609	8.199	1	0.00419	3.470	1	0.0625

Table S3 - outputs of the Likelihood Ratio Tests conducted on all generalised linear mixed effects models examining the explanatory variable cross and its interaction with timing block. These models include data from only conspecific crosses.

Response Variable	Model	AICc	Weighted AIC	Model Selected
Mating Attempt Occurrence	Female and Male Strains	452.42	1.00	Female and Male Strains
	Mating Type	468.78	0.00	
Latency to First Mating Attempt	Female and Male Strains	2337.09	1.00	Female and Male Strains
	Mating Type	2352.83	0.00	
Female Kick	Female and Male Strains	214.28	0.24	Mating Type
	Mating Type	211.94	0.76	
Attempt Period	Female and Male Strains	2181.39	0.16	Mating Type
	Mating Type	2178.03	0.84	
Effortfulness	Female and Male Strains	294.96	0.69	Female and Male Strains
	Mating Type	296.58	0.31	

Table S4 - outputs of the Likelihood Ratio Tests conducted on all generalised linear mixed effects models examining the explanatory variable mating type and its interaction with timing block. These models include data from both conspecific and heterospecific crosses.

Response Variable	Distribution	Mating Type			Timing Block			Mating Type * Timing Block		
		LRT	df	*p*	LRT	df	*p*	LRT	df	*p*
Mating Attempt Occurrence	Binomial	2.636	1	0.105	0.238	1	0.626	0.322	1	0.570
Latency to First Mating Attempt	Gamma	2.130	1	0.144	0.266	1	0.606	0.691	1	0.406
Female Kick	Binomial	21.663	1	3.250e-06	0.709	1	0.400	0.125	1	0.724
Attempt Period	Gamma	12.245	1	0.000466	0.026	1	0.872	0.0296	1	0.864
Effortful Attempt	Binomial	153.44	1	<2.2e-16	0.639	1	0.424	0.335	1	0.563

Table S5 –For each model where male strain, or its interaction with timing block, had a significant impact on the response variable, we also formed a model examining the effect of mating type. we determined which model explained the data better using AICc and Weighted AIC, as summarised.
