# Supplementary material for: A tiger is not always a satyr: role of male mating behaviour in interspecific mating interactions between Aedes aegypti and Aedes albopictus
Source: Parasit Vectors. 2026 Mar 13;19:238. doi: 10.1186/s13071-026-07267-7 (PMC13227695; doi:10.1186/s13071-026-07267-7)
Supplement: Supplementary file 2 — Additional file 2: Text S2. Additional methods. [file 13071_2026_7267_MOESM2_ESM.docx]

**Supplementary Information - Methods**

**Re-partitioning Time**

Aedes activity levels vary in a predictable way diurnally (1-3), and thus during experiments we recorded the timing of each cage observation so that we could include timing as a fixed effect in the models. We recorded timing in approximately 1.5 hour blocks. However, partitioning timing so finely resulted in some timing blocks having a very high standard error in some models. For instance, this occurred in models of count data, when there were some timing blocks where all observations were zero.

To solve this problem, we re-partitioned time. To decide on the size of the partitions, we examined the impact of timing block in isolation on all mating behaviours. We examined this separately for Ae. albopictus and Ae. aegypti males, as there may be differences in how time affects activity between these species.

We only found significant differences between timing blocks in the morning, and timing blocks in the afternoon, so we grouped as such. This grouping of timing block makes theoretical sense, as previous studies have shown differences in the activity levels of Aedes between the morning and afternoon (1-3).

**Model selection**

When analysing data from both conspecific and heterospecific crosses, for each response variable we formed a model examining the impact of female strain, male strain, and their interactions with timing block. Male strain incorporates two pieces of information – strain specific differences and mating type (conspecific/heterospecific), as each female is crossed with a conspecific of the same strain, and a heterospecific. Thus, when we found a significant effect of male strain, we formed another model to examine the impact of mating type (conspecific/heterospecific) and its interaction with timing block, on the response variable. We then used weighted AIC, using the package AICcmodavg (4), to determine whether the initial model, or the latter model is a better fit to the data. This allowed us to determine whether the observed differences are better explained by male strain or mating type. Only the results of the selected model were reported in the results section. Details of all models formed, and model selection are in the supplementary information (see supplementary Tables S2- S5).

When examining data from conspecific crosses, for each response variable we formed a model examining the impact of cross, timing block and their interaction. This is because, in conspecific crosses, mating only occurs between males and females of the same strain.

**References**

1. Gentile C, Rivas GB da S, Lima JB, Bruno RV, Peixoto AA. **Circadian clock of *Aedes aegypti*: effects of blood-feeding, insemination and RNA interference**. *Mem Inst Oswaldo Cruz.* 2013; **108**:80–7.
2. Egid BR, Coulibaly M, Dadzie SK, Kamgang B, McCall PJ, Sedda L, Toe KH, Wilson AL. **Review of the ecology and behaviour of *Aedes aegypti* and *Aedes albopictus* in Western Africa and implications for vector control**. *Curr Res Parasitol Vector-Borne Dis.* 2022; **2**:100074.
3. Araripe LO, Bezerra JRA, Rivas GBDS, Bruno RV. **Locomotor activity in males of *Aedes aegypti* can shift in response to females’ presence**. *Parasit Vectors.* 2018; **11**:254
4. Mazerolle MJ (2023). **AICcmodavg: Model selection and multimodel inference based on (Q)AIC(c).** R package version 2.3.3, https://cran.r-project.org/package=AICcmodavg.
